# Low-dose thiamine supplementation of lactating Cambodian mothers improves human milk thiamine concentrations: a randomized controlled trial

**DOI:** 10.1093/ajcn/nqab052

**Published:** 2021-04-07

**Authors:** Jelisa Gallant, Kathleen Chan, Tim J Green, Frank T Wieringa, Shalem Leemaqz, Rem Ngik, Jeffrey R Measelle, Dare A Baldwin, Mam Borath, Prak Sophonneary, Lisa N Yelland, Daniela Hampel, Setareh Shahab-Ferdows, Lindsay H Allen, Kerry S Jones, Albert Koulman, Damon A Parkington, Sarah R Meadows, Hou Kroeun, Kyly C Whitfield

**Affiliations:** Department of Applied Human Nutrition, Mount Saint Vincent University, Halifax, NS Canada; Department of Applied Human Nutrition, Mount Saint Vincent University, Halifax, NS Canada; South Australian Health and Medical Research Institute, Adelaide, SA, Australia; School of Medicine, University of Adelaide, Adelaide, SA, Australia; UMR-204, Institut de recherche pour le développement, UM/IRD/SupAgro, Montpellier, France; South Australian Health and Medical Research Institute, Adelaide, SA, Australia; Helen Keller International Cambodia, Phnom Penh, Cambodia; Department of Psychology, University of Oregon, Eugene, OR, USA; Department of Psychology, University of Oregon, Eugene, OR, USA; National Subcommittee for Food Fortification, Cambodia Ministry of Planning, Phnom Penh, Cambodia; National Nutrition Programme, Maternal and Child Health Centre, Cambodia Ministry of Health, Phnom Penh, Cambodia; South Australian Health and Medical Research Institute, Adelaide, SA, Australia; School of Public Health, University of Adelaide, Adelaide, SA, Australia; USDA, ARS Western Human Nutrition Research Center, University of California, Davis, CA, USA; Department of Nutrition, University of California, Davis, CA, USA; USDA, ARS Western Human Nutrition Research Center, University of California, Davis, CA, USA; Department of Nutrition, University of California, Davis, CA, USA; USDA, ARS Western Human Nutrition Research Center, University of California, Davis, CA, USA; Department of Nutrition, University of California, Davis, CA, USA; NIHR BRC Nutritional Biomarker Laboratory, MRC Epidemiology Unit, University of Cambridge, Cambridge, United Kingdom; NIHR BRC Nutritional Biomarker Laboratory, MRC Epidemiology Unit, University of Cambridge, Cambridge, United Kingdom; NIHR BRC Nutritional Biomarker Laboratory, MRC Epidemiology Unit, University of Cambridge, Cambridge, United Kingdom; NIHR BRC Nutritional Biomarker Laboratory, MRC Epidemiology Unit, University of Cambridge, Cambridge, United Kingdom; Helen Keller International Cambodia, Phnom Penh, Cambodia; Department of Applied Human Nutrition, Mount Saint Vincent University, Halifax, NS Canada

**Keywords:** thiamine (vitamin B1), supplementation, human milk, ThDP, ETKac

## Abstract

**Background:**

Infantile beriberi–related mortality is still common in South and Southeast Asia. Interventions to increase maternal thiamine intakes, and thus human milk thiamine, are warranted; however, the required dose remains unknown.

**Objectives:**

We sought to estimate the dose at which additional maternal intake of oral thiamine no longer meaningfully increased milk thiamine concentrations in infants at 24 wk postpartum, and to investigate the impact of 4 thiamine supplementation doses on milk and blood thiamine status biomarkers.

**Methods:**

In this double-blind, 4–parallel arm randomized controlled dose–response trial, healthy mothers were recruited in Kampong Thom, Cambodia. At 2 wk postpartum, women were randomly assigned to consume 1 capsule, containing 0, 1.2 (estimated average requirement), 2.4, or 10 mg of thiamine daily from 2 through 24 weeks postpartum. Human milk total thiamine concentrations were measured using HPLC. An Emax curve was plotted, which was estimated using a nonlinear least squares model in an intention-to-treat analysis. Linear mixed-effects models were used to test for differences between treatment groups. Maternal and infant blood thiamine biomarkers were also assessed.

**Results:**

In total, each of 335 women was randomly assigned to1 of the following thiamine-dose groups: placebo (*n = *83), 1.2 mg (*n = *86), 2.4 mg (*n = *81), and 10 mg (*n = *85). The estimated dose required to reach 90% of the maximum average total thiamine concentration in human milk (191 µg/L) is 2.35 (95% CI: 0.58, 7.01) mg/d. The mean ± SD milk thiamine concentrations were significantly higher in all intervention groups (183 ± 91, 190 ± 105, and 206 ± 89 µg/L for 1.2, 2.4, and 10 mg, respectively) compared with the placebo group (153 ± 85 µg/L; *P* < 0.0001) and did not significantly differ from each other.

**Conclusions:**

A supplemental thiamine dose of 2.35 mg/d was required to achieve a milk total thiamine concentration of 191 µg/L. However, 1.2 mg/d for 22 wk was sufficient to increase milk thiamine concentrations to similar levels achieved by higher supplementation doses (2.4 and 10 mg/d), and comparable to those of healthy mothers in regions without beriberi. This trial was registered at clinicaltrials.gov as NCT03616288.

## Introduction

Thiamine, also known as vitamin B1, is a water soluble vitamin essential in energy metabolism, neuronal functioning, and cognitive development (1). Thiamine deficiency can occur at any life stage but is most serious in infancy as it can lead to infantile beriberi, and even death within hours of the onset of signs and symptoms (2). Recent evidence suggests that milder forms of infantile thiamine deficiency, not serious enough to cause beriberi, can lead to long-term cognitive impairments (3, 4). Thiamine deficiency is still reported throughout South and Southeast Asia (5) and is largely attributed to a diet high in polished white rice (6). Not only is rice thiamine poor, but a high-carbohydrate diet also increases thiamine requirements (7, 8). Lactating mothers consuming this diet produce thiamine-poor milk, putting their exclusively breastfed infants at risk of cognitive impairment (9) or infantile beriberi (10). Thus, to prevent infantile thiamine deficiency, milk thiamine content must be improved through interventions targeting mothers of at-risk populations during the perinatal period (11).

The aim of this study was to estimate the maternal dose of oral thiamine at which additional thiamine intake no longer meaningfully increases human milk total thiamine concentrations at 24 wk postpartum. In addition, we aimed to examine the impact of longer-term maternal intake of various doses of oral thiamine on maternal and infant thiamine biomarkers: human milk total thiamine concentrations, whole blood thiamine diphosphate (ThDP) concentrations, and erythrocyte transketolase activity coefficients (ETKac). Once the optimal dose of thiamine among lactating women is established, better informed public health programs such as fortification of a staple food or condiment or addition of thiamine into perinatal multiple micronutrient supplements can be developed and implemented.

## Methods

### Study design

The full study protocol is published elsewhere (12). This was a community-based, double-blind, 4–parallel arm randomized controlled dose–response trial that took place in Kampong Thom, Cambodia, between September 2018 and May 2019. Women were recruited from 8 health centers selected in collaboration with the Cambodian Ministry of Health. Ethical approval was obtained from the National Ethics Committee for Health Research, Cambodia (112/250NECHR), Mount Saint Vincent University Research Ethics Board, Canada (2017–141), and the University of Oregon Institutional Review Board, United States (07052018.008).

### Participants

Women were recruited through antenatal care visits and entered into the study after delivery. To participate, women had to meet the following eligibility criteria (12): be a mother (18–45 y) whose most recent pregnancy was normal, with a singleton infant born without complications. The mother had to live in Kampong Thom province (and not intend to move within 6 mo), intend to exclusively breastfeed for 6 mo, have not consumed any thiamine-containing supplements in the 4 preceding mo, and not currently be participating in any nutrition programs beyond normal care. The mother must have indicated her willingness to consume 1 capsule daily from 2 through 24 wk postpartum and to have biological samples collected from herself and her infant throughout the study. Women provided written informed consent for themselves and their infant.

### Randomization and masking

Women were randomly assigned at 2 wk postpartum to 1 of 4 treatment groups [placebo: 0 mg, estimated average requirement (EAR): 1.2 mg; double the EAR: 2.4 mg; and a positive control group: 10 mg), and asked to consume 1 capsule daily between 2 and 24 wk postpartum. These opaque gelatin capsules were identical in size and shape, but contained varying amounts of thiamine as thiamine hydrochloride and cellulose as a filler. Capsules were formulated and compounded at the Quinpool Wellness Centre in Halifax, Nova Scotia, Canada. Thiamine content was assessed by an independent laboratory (USANA) before distribution to participants and periodically throughout the study; only minor losses were noted after experimental 1-mo storage in rural Cambodian households (97—106% of expected thiamine content).

Capsules were packaged in identical 14-d blister packs with uninformative printed alphanumeric treatment code labels. Capsule counts were completed by field staff who visited the participants’ homes fortnightly to provide a new blister pack. Women also received daily short message service (SMS) text and weekly phone call reminders; however, if a woman missed a capsule, she was advised not to “make up” the missing dose by taking it on a subsequent day.

A computer-generated randomization schedule was prepared by the study statisticians using ralloc.ado in Stata version 15.1 with blinded treatment code labels. Randomly permuted blocks of size 8 within health center strata were used to assign participants to 1 of 8 treatment codes in the ratio 1:1:1:1:1:1:1:1 (2 treatment codes per treatment group to assist with blinding). The treatment group mapping to each treatment code was performed by an independent scientist. The treatment codes were kept in sealed opaque envelopes labeled with the study ID and opened by the research assistants when a participant was enrolled in the study. Participants, research assistants, and study investigators were blinded to the randomly assigned groups. Data analysts were necessarily unblinded during the analysis since a blinded analysis is not possible for estimating dose–response curves.

### Data and biological sample collection

Participant demographic, socioeconomic, health, and dietary information were collected by healthcare workers at delivery, and by trained field workers at baseline (2 wk postpartum), midline (12 wk postpartum), and endline (24 wk postpartum). Using calibrated instruments and standard protocols (13), mother and infant anthropometric measurements were collected at all timepoints (see **Supplementary Table 1**).

All biological samples were collected in women's homes, or a central village meeting space. Human milk samples were collected at 2, 4, 12, and 24 wk postpartum using a battery powered single-breast pump (Swing Breast pump, Medela). One single, full breast expression was collected from the breast that participants self-identified as more “full.” Nonfasting venous blood samples were collected from mothers (2 and 24 wk) and infants (24 wk) into evacuated tubes containing EDTA (Vacuette K3E, Greiner Bio-One) by trained phlebotomists. Samples were transported to the field lab in Kampong Thom in iceboxes within 5 h of collection. Human milk and whole blood samples were divided into aliquots. Blood samples were then centrifuged at 3000 rpm (approximately 600 x g) for 15 min at 4°C, the plasma and buffy coat were removed, and erythrocytes were washed 3 times with PBS (Sigma Life Science). All samples were stored at −20°C for up to 10 d before being moved to −80°C freezers. Samples were batch shipped on dry ice to collaborating laboratories for analysis.

Human milk thiamine concentrations were assessed (14) at the USDA, ARS Western Human Nutrition Research Center, using an Agilent 1200 HPLC with fluorescence detector. Total thiamine concentrations were calculated based on molecular weights: total thiamine = free thiamine + (thiamine monophosphate × 0.871) + (thiamine diphosphate × 0.707).

ETKac and whole blood ThDP concentrations were analyzed at the NIHR Nutritional Biomarker Laboratory. Whole blood ThDP was measured based on a method published by Zhang et al. (15) using a Waters 2695 Alliance HPLC with a fluorescence detector. Raw ThDP values were also corrected for hematocrit (1), measured manually in the field with capillary hematocrit tubes (LW Scientific ZIPCombo Centrifuge and EZ Reader Microhematocrit Reader). Quality control (QC) for the ThDP assay was achieved with 3 QC materials measured in duplicate with each batch (*n *= 36); 2 Chromsystems QCs (Chromsystems Instruments & Chemicals GmbH) and 1 in-house control prepared from commercially supplied whole blood (BioIVT). The QCs had mean ThDP concentrations of 41,151 and 166 nmol/L and CVs of 9.8%, 10.5%, and 7.3%, respectively.

For ETKac, basal and activated (with the addition of exogenous ThDP) activities of the ThDP-dependent enzyme transketolase were measured in duplicate in washed erythrocyte hemolysates. The assay was performed in 96-well plates, and the rate of oxidation of NADH (the final product of the reaction) was measured by monitoring the decrease in absorbance at 340 nm (Thermo Multiskan FC, Thermo Fisher Scientific) as described by Jones et al. (16). The ETKac is the ratio of activated and basal activities. QC material for the ETKac assay was prepared in-house from whole blood and run in duplicate in each batch (*n *= 47); CVs were 2.8%, 3.4%, and 2.9%, respectively, at ETKacs of 1.06, 1.15, and 1.19. The ETKac indicates the degree of ThDP saturation of transketolase; an ETKac ratio of 1.0 indicates complete basal ThDP saturation, while a higher ETKac is indicative of poorer thiamine status (17).

Risks of deficiency with ETKac are most commonly defined as low risk, <1.15; moderate risk 1.15–1.25; and high risk, >1.25 (1). Although there are no widely agreed upon cutoffs for thiamine status as measured by ThDP (18), a whole blood ThDP cutoff of <95 nmol/L (19), which aligns with the reference interval set by Lu and Frank (20), was used to categorize mothers with low thiamine status. There are currently no cutoffs for thiamine in human milk.

### Outcomes

The primary outcome of this study was human milk total thiamine concentration at 24 wk postpartum and the primary objective was to estimate the dose on the dose–response curve where additional maternal intake of oral thiamine no longer meaningfully increased human milk total thiamine concentration (defined as the dose that reached 90% of the maximum average concentration) at 24 wk postpartum. Additionally, we aimed to assess differences between the 4 randomly assigned groups in human milk total thiamine and 2 biomarkers of thiamine status, whole blood ThDP and ETKac. No tolerable upper limit has been established for thiamine (17, 21); as such, no Serious Adverse Events Committee or Data Safety Monitoring Board were deemed necessary (12). During weekly calls and fortnightly visits, field staff asked mothers about signs of infantile beriberi (1); participants were also given mobile phones and top-ups so they could call field staff in case of suspected beriberi or other concerns with the study. There were no adverse events related to the study; 1 infant died during the study, but the attending physician confirmed that the death was not study related.

### Statistical analysis

The statistical analysis plan is available at clinicaltrials.gov, identifier NCT03616288. Briefly, a total sample size of 192 women was required to detect a clinically meaningful difference of 40 µg/L in human milk total thiamine concentration with an estimated SD of 43 µg/L (11), 90% power, and ≥20% attrition using a 2-sided alpha of 0.0083 for each of the 6 pairwise comparisons between the 4 treatment groups in order to control the familywise error rate at the 0.05 level using a Bonferroni adjustment for multiple comparisons. Recruitment of 320 women (80 per group) was planned to account for uncertainty in the assumed values.

Since a blinded analysis is not possible for estimating dose response curves, unblinded treatment codes were included in the database following database lock. Descriptive statistics were computed for demographic and health information. The primary analyses were performed according to the randomly assigned treatment group regardless of compliance (intention to treat). A secondary “per-protocol” analysis was performed in the subset of women who consumed ≥80% of the randomized capsules over the study period. The parameters of the Emax dose–response curve were estimated using nonlinear least squares models. A 3-parameter Emax model (assuming the Hill factor equals 1) was fitted instead of the planned 4-parameter model due to convergence issues, and analyses were based on raw rather than imputed data due to the complexity of the models and the wide CIs obtained using the raw data. The dose that achieved 90% of the maximum average concentration was estimated from the fitted curve with bootstrapped 95% CIs.

Linear mixed-effects models were used to test for differences in means between treatment groups while accounting for repeated measurements and adjusting for randomization strata and biochemical data at 2 wk postpartum in the primary adjusted analyses; unadjusted analyses were also performed. Missing data were addressed using multiple imputation by treatment group to create 100 complete data sets for analysis, and a sensitivity analysis was performed on the raw data (22). All statistical analyses were performed using R version 3.6.3 (R Foundation for Statistical Computing). Emax curves and mixed-effects models were fitted using the nlme package (23), and the investr package (24) was used for dose estimation from the fitted Emax curves.

## Results

**FIGURE 1:**
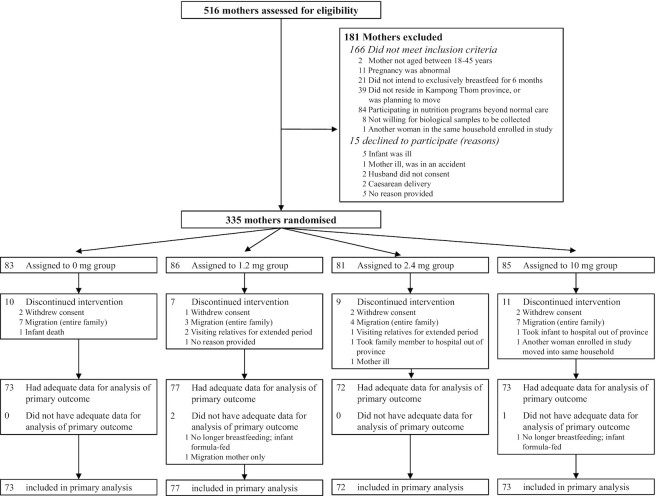
Trial profile for mother–infant dyads in Kampong Thom, Cambodia.

Between 28 August and 24 December 2018, 516 women were screened for eligibility, and 335 mother–infant dyads met the eligibility criteria, agreed to participate in the study, and were randomly assigned to 1 of the 4 treatment groups (placebo, *n *= 83; or thiamine dose 1.2 mg, *n *= 86; 2.4 mg, *n *= 81; of 10 mg, *n *= 85). The trial profile is shown in **Figure 1**. At 24 wk postpartum, milk samples were available for analysis from 295 mothers. The main reason for loss to follow-up was migration from Kampong Thom province.

The women's mean age was 28 y, two-thirds of the women were multiparous, and all women study participants self-reported to be of Khmer ethnicity (**Table 1**). Nearly half of the women and their husbands had completed primary education. Household size was on average 4 people, and 45% of participants’ households fell within the lowest 2 national wealth quintiles (25). Compliance was high, with 89% of women consuming ≥80% of their capsules.

**FIGURE 3:**
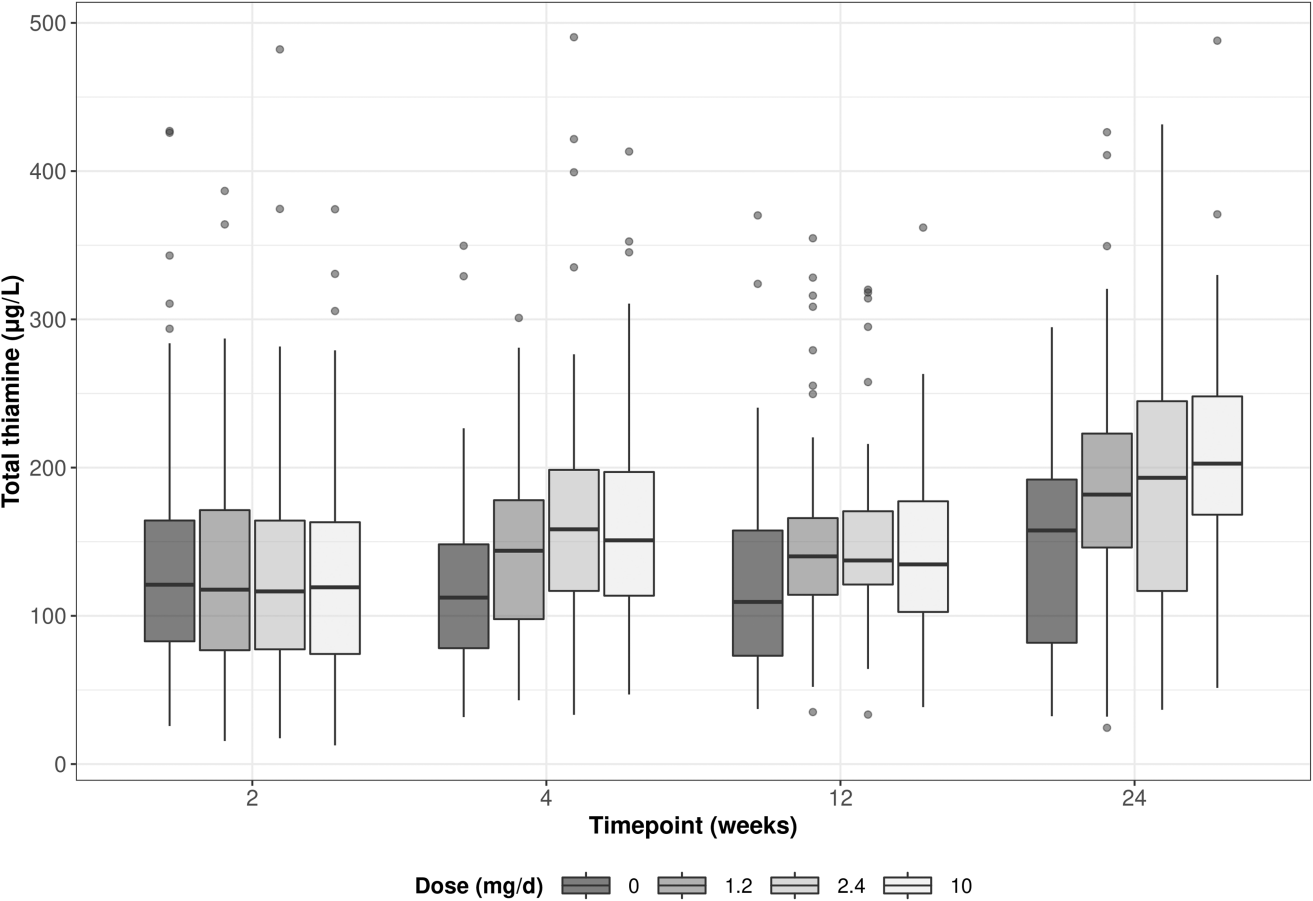
Boxplots of human milk total thiamine concentration by oral thiamine administration among lactating Cambodian women at 2 to 24 wk postpartum.

**TABLE 1:** Baseline characteristics of randomly assigned participants, by treatment group^1^

	Treatment group and thiamine dose			
	Placebo, 0 mg (*n *= 83)	1.2 mg (*n *= 86)	2.4 mg (*n *= 81)	10 mg (*n *= 85)
Mother				
Age, y	28.3 ± 6.1	27.9 ± 6.7	28.1 ± 6.1	28.1 ± 5.9
Parity, multiparous	54 (65%)	54 (63%)	58 (72%)	64 (75%)
Ethnicity, Khmer	83 (100%)	86 (100%)	81 (100%)	85 (100%)
Marital status, married	79 (95%)	86 (100%)	81 (100%)	84 (99%)
Education				
None	10 (12%)	8 (9%)	13 (16%)	9 (11%)
Primary (1–6 y)	43 (52%)	37 (43%)	40 (49%)	41 (48%)
Lower secondary (7–9 y)	16 (19%)	29 (34%)	19 (24%)	19 (22%)
Upper secondary (10–12 y)	12 (15%)	9 (11%)	8 (10%)	14 (17%)
Higher education	2 (2%)	3 (3%)	1 (1%)	2 (2%)
Occupation				
Homemaker	42 (51%)	41 (48%)	40 (49%)	40 (47%)
Farmer	19 (23%)	27 (31%)	23 (28%)	25 (29%)
Unemployed	6 (7%)	3 (3%)	8 (10%)	6 (7%)
Seller	4 (5%)	9 (11%)	4 (5%)	5 (6%)
Other	12 (14%)	6 (7%)	6 (8%)	9 (11%)
Chews betel nut/betel leaf				
Daily	4 (5%)	1 (1%)	1 (1%)	1 (1%)
Occasionally	16 (19%)	7 (8%)	15 (19%)	15 (18%)
Never	63 (76%)	78 (91%)	65 (80%)	69 (81%)
Typically experiences diarrhea	2 (2%)	3 (4%)	3 (4%)	1 (1%)
Household				
Husband education				
None	10 (12%)	9 (10%)	9 (11%)	10 (12%)
Primary (1–6 y)	42 (51%)	37 (43%)	39 (48%)	33 (39%)
Lower secondary (7–9 y)	21 (25%)	24 (28%)	23 (28%)	29 (34%)
Upper secondary (10–12 y)	5 (6%)	13 (15%)	8 (10%)	8 (9%)
Higher education	5 (6%)	3 (3%)	2 (3%)	5 (6%)
Household size, number of people	3.7 ± 1.7	3.6 ± 1.8	4.0 ± 2.1	4.1 ± 2.0
Annual household income, US$	1800 (950–3000)	2050 (963–3500)	1600 (1000–3000)	2000 (1200–3500)
Wealth index score^2^				
Poorest	22 (27%)	12 (15%)	21 (26%)	25 (29%)
Second	16 (19%)	14 (16%)	20 (25%)	19 (22%)
Middle	26 (31%)	31 (36%)	24 (30%)	27 (32%)
Fourth	14 (17%)	20 (23%)	11 (13%)	9 (11%)
Wealthiest	5 (6%)	8 (10%)	5 (6%)	5 (6%)
Infant				
Sex, female	43 (52%)	43 (50%)	33 (41%)	42 (49%)
Length at 2 wk postnatal, cm	50.9 ± 1.9	50.7 ± 2.1	50.7 ± 2.0	50.7 ± 1.9
Weight at 2 wk postnatal, kg	3.5 ± 0.5	3.4 ± 0.5	3.4 ± 0.4	3.4 ± 0.5
Head circumference at 2 wk postnatal, cm	35.0 ± 1.2	34.7 ± 1.1	34.6 ± 1.4	34.7 ± 1.1

1 Data are means ± SDs or *n* (%), except household income data, shown as medians (IQRs). Percentages may not add to 100% due to rounding.

2 Wealth index quintiles calculated based on the Demographic Health Survey Program guidelines (USAID); Cambodian Wealth Index scores developed using the 2014 Cambodian Demographic and Health Survey.

**FIGURE 2:**
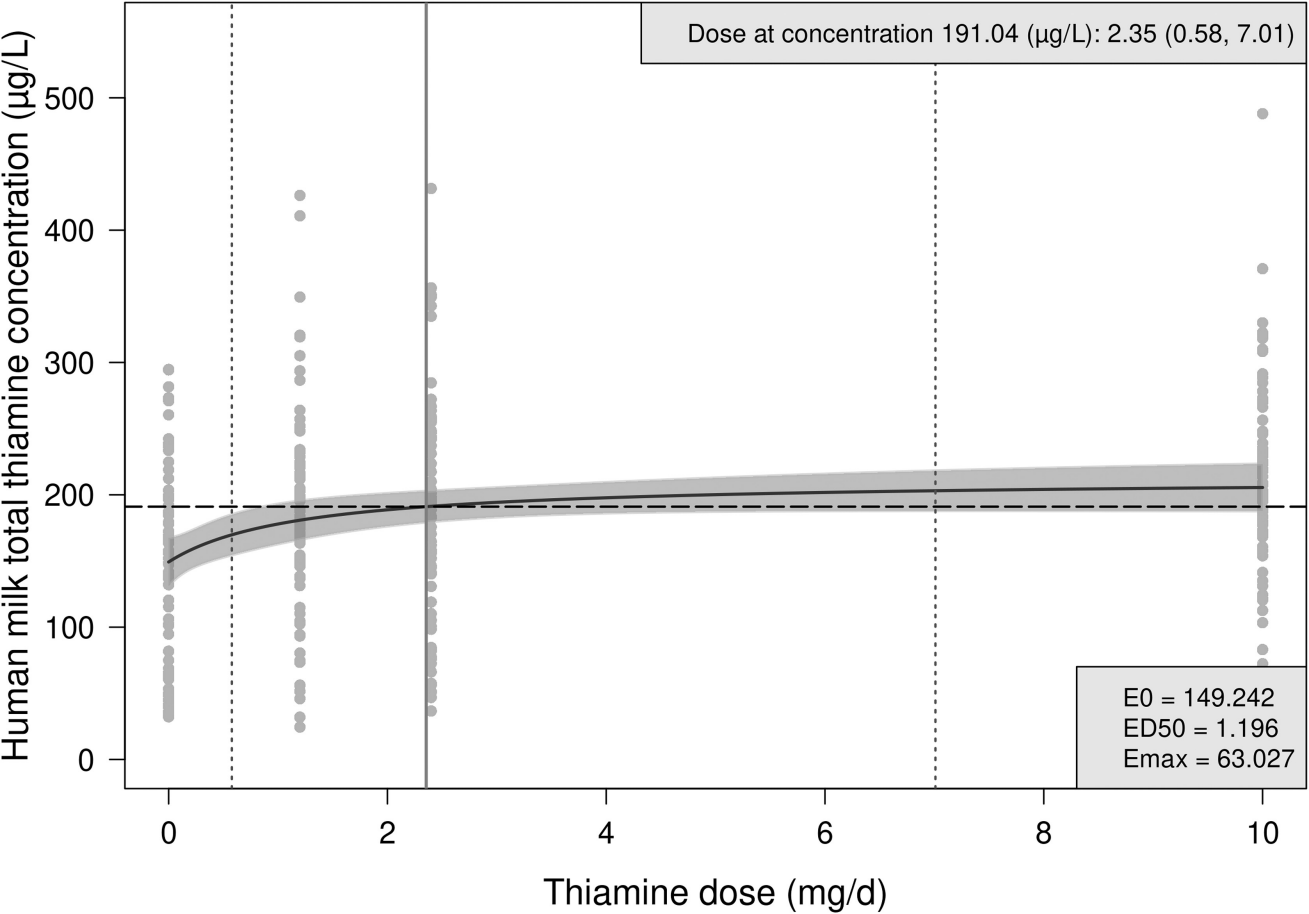
Emax dose–response curve for human milk total thiamine concentration by oral thiamine administration among lactating Cambodian woman at 24 wk postpartum. The solid black line indicates the estimated average human milk total thiamine concentration across doses based on the Emax model [${\rm concentration} = {\rm E0} + {\rm Emax}( {\rm dose}/( {\rm ED}50 + {\rm dose}))]$, with 95% confidence bands shaded in grey. The vertical, solid grey line indicates the estimated dose at 90% of the maximum average human milk total thiamine concentration (i.e., at 191.04 μg/L), with stratified bootstrapped 95% CIs shown in vertical gray dotted lines. Fitted Emax model parameters are shown in the bottom-right box. ED50, dose that produces 50% of the maximum effect of supplementation; Emax, maximum effect of supplementation; E0, baseline concentration without thiamine supplementation.

**Figure 2** shows the Emax dose–response curve for human milk total thiamine concentrations at 24 wk postpartum. A mean dose of maternal thiamine of 2.35 (95% CI: 0.58, 7.01) mg/d was estimated to be required to reach 90% of the maximum average thiamine concentration in human milk in this study, which was 191 µg/L.

At 24 wk postpartum, mean ± SD human milk total thiamine concentrations ranged from 153 ± 85 µg/L in the placebo group to 206 ± 89 µg/L in the 10-mg group (**Table 2** and **Figure 3**). In both unadjusted and adjusted analyses and across all time points, the average human milk total thiamine concentration was significantly higher in all 3 of the thiamine groups (1.2, 2.4, and 10 mg) than in the placebo group (*P* < 0.0001; **Table 3**). Estimated differences between the thiamine groups were all small (<10 µg/L) and none of the thiamine groups differed significantly from one another (Table 3).

**TABLE 2:** Thiamine biomarkers in human milk and blood samples, by treatment group^1^

		Treatment group and thiamine dose			
	Raw vs. corrected	Placebo (0 mg) *n *= 83	1.2 mg *n *= 86	2.4 mg *n *= 81	10 mg *n *= 85
Human milk total thiamine concentrations (µg/L)^2^					
2 wk postpartum		135.5 ± 77.7	129.3 ± 71.4	126.2 ± 77.2	125.4 ± 72.3
4 wk postpartum		118.8 ± 59.8	145.1 ± 55.7	168.0 ± 76.9	162.0 ± 76.6
12 wk postpartum		119.0 ± 69.8	149.8 ± 63.5	148.4 ± 62.7	142.2 ± 62.0
24 wk postpartum		152.5 ± 84.8	183.2 ± 90.6	190.1 ± 105.1	205.6 ± 89.4
Whole blood ThDP, nmol/L					
Mother					
2 wk postpartum					
	Raw^3^	72.9 ± 28.9	68.9 ± 25.2	68.6 ± 25.4	71.2 ± 21.0
	Corrected^4^	200.3 ± 69.4	197.1 ± 60.3	179.7 ± 79.8	197.3 ± 52.2
Low status (<95)	Raw^3^	82%	85%	84%	87%
24 wk postpartum					
	Raw^3^	80.4 ± 21.8	96.9 ± 22.9	98.8 ± 21.7	111.1 ± 25.2
	Corrected^4^	222.2 ± 62.9	264.1 ± 72.1	261.7 ± 62.2	298.3 ± 74.7
Low status (<95)	Raw^3^	68%	43%	41%	24%
Infant					
24 wk postnatal	Raw^3^	57.3 ± 14.1	73.9 ± 17.2	72.8 ± 19.5	73.2 ± 17.4
	Corrected^4^	180.9 ± 58.4	237.4 ± 75.0	222.9 ± 73.5	228.2 ± 61.5
ETKac					
Mother					
2 wk postpartum		1.15 ± 0.08	1.16 ± 0.10	1.16 ± 0.10	1.17 ± 0.09
Low risk (<1.15)		58%	56%	53%	49%
Moderate risk (1.15–1.25)		34%	30%	36%	33%
High risk (>1.25)		7%	14%	11%	18%
24 wk postpartum		1.20 ± 0.08	1.15 ± 0.06	1.14 ± 0.07	1.12 ± 0.06
Low risk (<1.15)		29%	54%	60%	73%
Moderate risk (1.15–1.25)		46%	40%	36%	22%
High risk (>1.25)		25%	7%	4%	5%
Infant					
24 wk postnatal		1.18 ± 0.10	1.14 ± 0.07	1.14 ± 0.07	1.12 ± 0.07
Low risk (<1.15)		38%	54%	60%	76%
Moderate risk (1.15–1.25)		37%	41%	35%	21%
High risk (>1.25)		24%	5%	4%	3%

1 Values are means ±SDs or percentages based on imputed data unless otherwise indicated. Percentages may not add to 100% due to rounding. Results of statistical comparisons between treatment groups are presented in Table 3. ETKac, erythrocyte transketolase activity coefficient; ThDP, thiamine diphosphate concentrations.

2 Human milk total thiamine concentrations are calculated as: free thiamine + (thiamine monophosphate × 0.871) + (thiamine diphosphate × 0.707).

3 Unimputed data reported as raw ThDP measurements were not included in the analysis.

4 Values corrected for hematocrit.

**TABLE 3:** Mean differences in human milk and blood thiamine biomarkers, between treatment groups^1^

	Unadjusted mean difference, µg/L	*P* value^2^	Adjusted mean difference,^3^ µg/L	*P* value^2^
Human milk total thiamine concentration,^4^ µg/L				
1.2 mg − placebo (0 mg)	29.05 (12.17, 45.94)	<0.0001	30.09 (13.30, 46.88)	<0.0001
2.4 mg − placebo (0 mg)	38.66 (19.88, 57.45)	<0.0001	39.84 (21.21, 58.47)	<0.0001
2.4 mg − 1.2 mg	9.61 (−8.32, 27.54)	0.5	9.75 (−7.99, 27.49)	0.5
10 mg − placebo (0 mg)	37.25 (18.71, 55.79)	<0.0001	38.65 (20.35, 56.95)	<0.0001
10 mg − 1.2 mg	8.19 (−9.30, 25.68)	0.6	8.56 (−8.85, 25.97)	0.6
10 mg − 2.4 mg	−1.42 (−20.93, 18.10)	1.0	−1.19 (−20.20, 17.93)	1.0
Overall		<0.0001		<0.0001
Whole blood ThDP,^5^ nmol/L				
Mother				
1.2 mg − placebo (0 mg)	43.03 (12.02, 74.05)	0.002	43.17 (12.64, 73.70)	0.002
2.4 mg − placebo (0 mg)	46.24 (13.17, 79.32)	0.002	46.01 (13.36, 78.66)	0.002
2.4 mg − 1.2 mg	3.21 (−30.28, 36.71)	1	2.84 (−30.14, 35.82)	1
10 mg − placebo (0 mg)	77.23 (44.79,109.67)	<0.0001	76.78 (44.81,108.76)	<0.0001
10 mg − 1.2 mg	34.19 (1.96, 66.42)	0.03	33.61 (1.93, 65.29)	0.03
10 mg − 2.4 mg	30.98 (−2.63, 64.59)	0.08	30.77 (−2.40, 63.95)	0.08
Overall		<0.0001		<0.0001
Infant				
1.2 mg − placebo (0 mg)	56.60 (24.50, 88.69)	<0.0001	57.01 (25.31, 88.72)	<0.0001
2.4 mg − placebo (0 mg)	42.04 (7.52, 76.55)	0.01	42.35 (8.00, 76.70)	0.009
2.4 mg − 1.2 mg	−14.56 (−50.71, 21.58)	0.7	−14.66 (−50.52, 21.20)	0.7
10 mg − placebo (0 mg)	47.31 (16.02, 78.61)	0.0007	46.83 (15.88, 77.78)	0.0007
10 mg − 1.2 mg	−9.29 (−40.65, 22.08)	0.9	−10.18 (−41.22, 20.86)	0.8
10 mg − 2.4 mg	5.28 (−27.80, 38.35)	1	4.48 (−28.22, 37.18)	1
Overall		<0.0001		<0.0001
ETKac^5^				
Mother				
1.2 mg − placebo (0 mg)	−0.05 (−0.08, −0.01)	0.004	−0.05 (−0.08, −0.01)	0.004
2.4 mg − placebo (0 mg)	−0.06 (−0.09, −0.02)	0.0002	−0.06 (−0.09, −0.02)	0.0002
2.4 mg − 1.2 mg	−0.01 (−0.04, 0.02)	0.7	−0.01 (−0.04, 0.02)	0.7
10 mg − placebo (0 mg)	−0.07 (−0.11, −0.04)	<0.0001	−0.07 (−0.11, −0.04)	<0.0001
10 mg − 1.2 mg	−0.03 (−0.06, 0.00)	0.1	−0.03 (−0.06, 0.00)	0.1
10 mg − 2.4 mg	−0.02 (−0.05, 0.02)	0.7	−0.02 (−0.05, 0.02)	0.6
Overall		<0.0001		<0.0001
Infant				
1.2 mg − placebo (0 mg)	−0.04 (−0.08, 0.01)	0.1	−0.04 (−0.08, 0.01)	0.1
2.4 mg − placebo (0 mg)	−0.04 (−0.09, 0.01)	0.1	−0.04 (−0.09, 0.01)	0.1
2.4 mg − 1.2 mg	−0.00 (−0.04, 0.03)	1	−0.00 (−0.04, 0.03)	1
10 mg − placebo (0 mg)	−0.06 (−0.10, −0.02)	0.003	−0.06 (−0.10, −0.02)	0.003
10 mg − 1.2 mg	−0.02 (−0.06, 0.01)	0.3	−0.02 (−0.06, 0.01)	0.3
10 mg − 2.4 mg	−0.02 (−0.06, 0.02)	0.6	−0.02 (−0.06, 0.02)	0.6
Overall		0.009		0.009

1 Data are mean differences (95% CIs) unless otherwise indicated. Results are based on analysis of 100 imputed datasets. ETKac, erythrocyte transketolase activity coefficient; ThDP, thiamine diphosphate concentrations.

2 Post hoc (Tukey) adjusted *P* values for multiple comparisons.

3 Adjustments: human milk concentrations, adjusted for human milk total thiamine at 2 wk and health center; ThDP, adjusted for health center (mother and infant) and maternal thiamine diphosphate concentrations (corrected for hematocrit) at 2 wk (mother only); ETKac, adjusted for health center (mother and infant) and erythrocyte transketolase activity coefficient at 2 wk (mother only).

4 Linear mixed-effects model was used to analyze the data at 4, 12, and 24 wk with a treatment by time point interaction model. Mean differences between treatment groups collapsed across all time points (4, 12, and 24 wk) are reported, as the treatment by time interaction *P* value was >0.05 and hence was dropped from the analysis model.

5 Linear regression was used to estimate the mean differences between treatment groups at 24 wk.

Maternal (2 and 24 wk postpartum) and infant (24 wk postnatal) whole blood ThDP concentrations are presented by treatment group in Table 2, and pairwise comparisons of mean differences at 24 wk are shown in Table 3. All thiamine groups had significantly higher maternal ThDP than the placebo group. The average maternal whole blood ThDP concentrations differed only between the 1.2- and 10-mg groups (*P* = 0.03); none of the other thiamine groups differed from one another (*P* > 0.05). Among infants, all thiamine groups had significantly higher ThDP than the placebo, and none of the thiamine groups differed significantly from each other.

ETKac is a functional biomarker of thiamine status, with higher activity coefficient ratios indicating poorer status (17). Table 2 shows maternal and infant ETKac ratios by treatment group, with pairwise comparisons in Table 3. Similar to the human milk results, average maternal ETKac ratios differed significantly between the placebo group and all 3 intervention groups (*P* < 0.05), and none of the thiamine groups differed significantly from one another. For infant ETKac, only the 10-mg and placebo groups differed significantly (*P* = 0.003).

Per protocol analyses produced similar results; see **Supplementary Tables 2 and 3**. Results from sensitivity analysis on raw data are similar to the results based on analysis of 100 imputed datasets.

## Discussion

Using the EMax curve (Figure 2) we estimated that a supplemental dose of 2.35 (95% CI: 0.58, 7.01) mg/d was required to achieve a mean human milk total thiamine concentration of 191 µg/L, 90% of the estimated maximum average concentration in our study. The CI associated with this estimated dose was much wider than expected, due to the large interindividual variations in milk thiamine concentrations seen at all doses. The SDs ranged from 85 to 105 µg/L at 24 wk, compared with an SD of 43 µg/L used in the sample size calculations based on data from a previous study in Cambodian women (11). This wide CI of the estimated dose limits the practical application of the Emax curve for accurate identification of an ideal daily intake at a population level. We cannot explain the larger SDs in the milk total thiamine concentrations seen in this study. However, large interindividual variation is not uncommon: ranges in milk thiamine concentrations among thiamine-replete American and Cambodian mothers (before and after supplementation) were 39–215, 29–124, and 125–280 μg/L, respectively (*n = *16 each; all values converted from nmol/L to μg/L) (26). This interindividual variability was also found in the Breast Milk Quality study, where interindividual variability accounted for between 70–85% of variability in milk thiamine concentrations over the 3-d study in Bangladesh (27). Given the apparent interindividual variability in biological capacity to move thiamine from maternal circulation into milk, the treatment group comparison results of the current study (Table 3) provide more useful information than the Emax curve (Figure 2) for determining the ideal, long-term maternal thiamine intake required to optimize milk thiamine concentrations, and in turn, infant biomarker status. While these results do not provide the same level of evidence as the primary trial analysis, they were from a prespecified secondary analysis of the primary outcome that was the basis of the sample size calculations and therefore provide valuable evidence for decision making.

We found that maternal thiamine supplementation of 1.2 mg/d for 22 wk was sufficient to increase human milk thiamine concentrations to similar levels achieved by higher supplementation doses (2.4 and 10 mg/d), and similar to milk concentrations of healthy mothers in regions where thiamine deficiency is not of concern. For example, thiamine-replete American mothers (*n *= 16; 6–28 wk postpartum) had median human milk thiamine concentrations of 173 μg/L (26), similar to the 1.2-mg group mean of 183 μg/L at 24 wk. Similarly, authors of a 1980 study investigating milk thiamine concentrations among *n = *57 Finnish mothers consuming a daily 2-mg thiamine supplement reported a mean ± SD of 199 ± 45 μg/L at 6 mo postpartum (28). Our results are also consistent with other interventions designed to increase human milk thiamine concentrations in areas where beriberi is prevalent. There was a significant increase in median human milk thiamine concentrations from 62 to 174 μg/L (*P* < 0.001) among Cambodian mothers (*n = *16; 1–7 mo postpartum) who consumed a daily 100-mg thiamine hydrochloride supplement (approximately 79 mg thiamine) for 5 d (26). Other Cambodian women (*n *= 87) who consumed thiamine-fortified fish sauce (2 or 8 g/L) ad libitum over a period of 6 mo in late pregnancy and early postpartum had higher mean human milk total thiamine concentrations of 207 and 177 µg/L, respectively, compared with women who consumed a control fish sauce containing no thiamine (144 µg/L) (11). The suggested dose of 1.2 mg/d based on the secondary analysis of the primary outcome is also well within the CI of 0.58–7.01 mg for the ideal dose based on the primary analysis.

In the current study, 48% of infants were still exclusively breastfed at 24 wk postpartum. However, all infants were still predominantly breastfed, with some limited introduction of water and rice porridge. To date, no cutoffs have been established to interpret the adequacy of thiamine in human milk. However, there is some evidence that that the adequate intake (AI) of 200 μg/d established for infants aged 0–6 mo (based on an estimated 780-mL daily consumption of milk containing 210 μg/L thiamine) (21) should be revisited, as very few milk samples globally have been found to meet this threshold (29). We similarly found that very few of the infants in this study were consuming milk containing ≥ 210 μg/L total thiamine: <38% at any timepoint or dose, suggesting that the current AI may not be a good proxy indicator of milk adequacy for thiamine.

As a functional indicator of status, ETKac is arguably the best thiamine biomarker (16, 17). However, measurement of ETKac is less common than measurement of ThDP, likely because of challenges with interassay precision and standardization (1), and simply because few laboratories currently have expertise in running this analysis. The mean ETKac of 1.18 among infants of mothers in the placebo group was significantly worse than the mean among infants in the 10-mg group (mean ETKac of 1.12), which differs from the infant ThDP results, according to which infants of mothers in all thiamine-receiving groups (1.2, 2.4, and 10 mg) had significantly higher ThDP than infants of mothers in the placebo group (Table 3). While this may indicate that a higher maternal dose of thiamine is needed to impact infant ETKac, other authors have suggested that assessment of basal transketolase activity alone could be a better indicator of thiamine status among infants (30). Further investigation of the use of transketolase biomarkers among infants, ideally with clinical indicators of beriberi, is warranted.

ThDP is a more commonly employed biomarker of thiamine status, likely because HPLC equipment is more ubiquitous in laboratories around the world; however, like ETKac, this method also suffers from a lack of standardization and matrix-matched reference material (1). Unfortunately there is a wide variety of cutoffs in the literature, and most such cutoffs were not established using clinical deficiency symptoms (18). As such, comparisons to whole blood ThDP values in thiamine-deficient and -replete populations are likely the best yardsticks. In the current study, at 24 wk the mean ThDP among placebo group women was 80 nmol/L, significantly lower than the thiamine-containing groups (1.2-, 2.4-, and 10-mg groups): 97, 99, and 111 nmol/L, respectively. Previously, Cambodian mothers have been found to have mean whole blood ThDP of 57 (31) and 58 nmol/L (26), while American “control” mothers in the same studies had ThDP of 126 (31), and 122 nmol/L (26). Infants have been previously shown to have higher ThDP than their mothers, and similarly, no valid cutoffs currently exist (18). However, the mean whole blood ThDP value in infants in the placebo group of our current study (57 nmol/L) is similar to previously reported values among unsupplemented Cambodian infants without symptoms of beriberi [mean 56 nmol/L (31); median 55 nmol/L (32)]. ThDP concentrations of well-nourished, thiamine-replete infants are largely unknown, as thiamine status is not often measured in national surveys, nor in high-income countries. More research is needed to set normative ThDP ranges in infants.

The EAR is the median daily intake value of a given micronutrient, consumed by any means (diet or supplement), that is estimated to meet the requirement of half the healthy individuals in a life-stage and sex group (33). The Institute of Medicine designed the thiamine EAR for lactating women in Canada and the United States with an assumed macronutrient distribution of 45–65% carbohydrates (33). More thiamine is likely required by Cambodian mothers due to their physically laborious lifestyles and higher carbohydrate intakes (1, 8). Although we did not collect dietary data, rates of household food insecurity are high (34), and dietary diversity is low in Cambodia [e.g., mean women's dietary diversity score of 4.7 of a possible 16 among factory workers (35)], with estimated thiamine intakes ranging from only 0.58 (36) to 0.88 mg/d (37). Our results suggest that a long-term 1.2-mg/d supplemental dose of thiamine, in addition to baseline dietary thiamine intakes, is sufficient to “top up” lactating mothers to an intake consistent with biomarker status of thiamine-replete Americans. The daily increase of 1.2 mg would likely benefit other mothers in other rice-consuming nations, such as Laos, Myanmar, and parts of India such as Assam (1).

It is important to consider vitamin dose delivery during program planning: supplementation delivers a bolus dose, whereas fortification usually delivers several small doses, with food, throughout the day. Saturation kinetics from previous thiamine absorption studies suggest that bolus doses >2.5 mg, at least among thiamine-replete individuals (38), may go largely unabsorbed (39). However, Donohue et al. recently reported similar increases in milk thiamine concentrations among Guatemalan mothers (4- to 6-mo postpartum) regardless of whether mothers consumed a 30-g portion of lipid-based nutrient supplement containing 2.8 mg thiamine as 1 bolus dose, or divided into three 10-g doses over 8 h (40). In the current study (bolus delivery), the lowest daily thiamine dose of 1.2 mg achieved milk concentrations that were similar to those from women consuming 2.4 mg and 10 mg daily, suggesting that a bolus 1.2-mg dose is sufficient for both supplementation and fortification program planning and may actually exceed the actual “top up” in thiamine required by rural Cambodian mothers.

A key strength of this study is that it is the first long-term (22 week) dose–response study of low-dose thiamine supplementation, as previous research explored therapeutic doses (e.g., 50 or 100 mg/d) over shorter timeframes (26, 41). Other strengths include employing 2 different biomarkers of thiamine status (ThDP and ETKac), low attrition, and strong compliance, likely due to the frequent (fortnightly) follow-ups with participants paired with daily SMS messages and weekly phone calls. However, our analysis is limited by the lack of interpretative criteria for human milk thiamine; clinically meaningful milk thiamine cutoffs must be developed to assess deficiency risk among mothers and infants. Another limitation is that the study started at 2 wk postpartum, so we do not have biochemical data from pregnancy. However, thiamine is preferentially sequestered to the fetus during the third trimester (42), and infant thiamine stores built up in utero are thought to last until ∼3 mo of age (18).

Based on this rigorous investigation of low-dose, long-term maternal thiamine supplementation, a supplemental dose of 2.35 (95% CI: 0.58, 7.01) mg/d achieved a mean human milk total thiamine concentration of 191 µg/L, which was 90% of the estimated maximum average concentration in our study. Group analyses suggest that 1.2 mg/d thiamine is sufficient to increase the biomarker status to levels consistent with those of thiamine-replete populations, which has the potential to prevent potentially fatal infantile beriberi. We encourage policy makers in regions where thiamine deficiency is thought to be a concern (e.g., Cambodia, Myanmar, Kiribati, Assam) (1, 5) to consider investing in programs such as the United Nations International Multiple Micronutrient Antenatal Preparation supplement (1.4 mg thiamine) for the perinatal period, or developing thiamine fortification programs that could reach lactating women and the wider population.

## Supplementary Material

nqab052_Supplemental_File

## Data Availability

Data described in the manuscript, code book, and analytic code will be made available upon request pending application and approval.

## Notes

Funded through the Bill & Melinda Gates Foundation and the New York Academy of Sciences (Opportunity ID OPP1176128). The funders were involved in the design of the study but had no role in the implementation, analysis, or interpretation of the data. We acknowledge USANA (Salt Lake City, UT) for the generous provision of laboratory analysis for the study of capsule thiamine hydrochloride content. JG and KC were supported through the Research Nova Scotia “Scotia Scholars Awards” and Canadian Institutes of Health Research Masters Graduate Scholarships. LNY was supported by an Australian National Health and Medical Research Council Early Career Fellowship (ID 1052388). KSJ, DAP, SRM, and AK are supported by the National Institute for Health Research (NIHR) Cambridge Biomedical Research Centre (IS-BRC-1215- 20014).

Supplemental Tables 1–3 are available from the “Supplementary data” link in the online posting of the article and from the same link in the online table of contents at https://academic.oup.com/ajcn/.

Abbreviations used: AI, adequate intake; ETKac, erythrocyte transketolase activity coefficients; EAR, estimated average requirement; QC, quality control; SMS, short message service; ThDP, thiamine diphosphate concentrations.
