# Supplementary material for: Low-dose thiamine supplementation of lactating Cambodian mothers improves human milk thiamine concentrations: a randomized controlled trial
Source: Am J Clin Nutr. 2021 Apr 7;114(1):90–100. doi: 10.1093/ajcn/nqab052 (PMC8246599; doi:10.1093/ajcn/nqab052)
Supplement: nqab052_Supplemental_File [file nqab052_supplemental_file.pdf]

Online Supplementary Material:
Gallant et al., Low dose thiamine supplementation of lactating Cambodian mothers optimizes human milk thiamine concentrations and would be expected to prevent beriberi: a randomized controlled trial

Supplementary Table 1: Anthropometric measurements of mothers and their infants <6 months[1]

	**Placebo (0 mg) *n*=83**	**1.2 mg *n*=86**	**2.4 mg *n*=81**	**10 mg *n*=85**
**Mother**				
2 weeks postpartum BMI, *kg/m²*	22.6 (3.2)	22.3 (2.7)	22.5 (2.8)	22.7 (3.1)
Underweight (≤ 18.5)	4 (5%)	2 (2%)	4 (5%)	3 (4%)
Normal (18.51-24.99)	61 (74%)	67 (78%)	66 (82%)	65 (77%)
Overweight (25-29.99)	17 (21%)	17 (20%)	9 (11%)	15 (18%)
Obese (≥ 30)	1 (1%)	0 (0%)	2 (3%)	2 (2%)
12 weeks postpartum BMI, *kg/m²*	22.8 (3.4)	22.7 (3.0)	22.9 (3.0)	22.8 (3.2)
Underweight (≤ 18.5)	6 (8%)	4 (5%)	5 (7%)	3 (4%)
Normal (18.51-24.99)	49 (65%)	59 (72%)	54 (72%)	59 (76%)
Overweight (25-29.99)	18 (24%)	19 (23%)	14 (19%)	14 (18%)
Obese (≥ 30)	2 (3%)	0 (0%)	2 (3%)	2 (3%)
24 weeks postpartum BMI, *kg/m²*	22.8 (3.7)	22.5 (3.4)	22.9 (3.4)	22.8 (3.5)
Underweight (≤ 18.5)	6 (8%)	10 (13%)	5 (7%)	3 (4%)
Normal (18.51-24.99)	49 (67%)	50 (64%)	51 (71%)	52 (70%)
Overweight (25-29.99)	16 (22%)	17 (22%)	14 (19%)	17 (23%)
Obese (≥ 30)	2 (3%)	1 (1%)	2 (3%)	2 (3%)
**Infant**				
Length-for-age (Z-score)				
2 weeks	-0.52 (0.98)	-0.66 (1.11)	-0.69(1.01)	-0.63 (1.01)
12 weeks[2]	-0.51 (1.29)	-0.73 (1.11)	-0.69 (0.83)	-0.60 (0.82)
24 weeks[3]	-0.68 (0.93)	-0.88 (0.01)	-1.03 (0.84)	-0.83 (0.88)
Weight-for-age (Z-score)				
2 weeks	-0.40 (0.95)	-0.50 (0.96)	-0.59 (0.88)	-0.58 (1.07)
12weeks[2]	-0.41 (0.96)	-0.60 (1.07)	-0.52 (0.90)	-0.40 (0.92)
24weeks[3]	-0.47 (0.95)	-0.77 (0.01)	-0.73 (0.91)	-0.60 (1.00)
Weight-for-length (Z-score)				
2 weeks	-0.30 (1.16)	-0.26 (1.15)	-0.42 (1.45)	-0.46 (1.34)
12weeks[2]	0.09 (1.44)	0.04 (1.16)	-0.19 (1.00)	0.15 (1.18)
24 weeks[3]	0.02 (1.07)	-0.22 (0.01)	-0.04 (1.03)	-0.04 (1.16)
Head circumference-for-age (Z-score)				
2 weeks	-0.59 (1.14)	-0.77 (0.98)	-0.81 (0.99)	-0.73 (0.96)
12 weeks[2]	-0.91 (0.91)	-1.14 (1.14)	-1.05 (0.89)	-1.02 (0.89)
24 weeks[3]	-0.77 (0.84)	-0.92 (0.01)	-1.15 (0.93)	-0.95 (0.93)

[1] Values are mean (SD) or n (%) based on raw data.
[2] Data collected at 12 weeks postnatal: placebo, *n*=75; 1.2 mg, *n*=82; 2.4 mg, *n*=75; 10 mg, *n*=78.
[3] Data collected at 24 weeks postnatal: placebo, *n*=73; 1.2 mg, *n*=79; 2.4 mg, *n*=72; 10 mg, *n*=74.

Online Supplementary Material:
Gallant et al., Low dose thiamine supplementation of lactating Cambodian mothers optimizes human milk thiamine concentrations and would be expected to prevent beriberi: a randomized controlled trial

Supplementary Table 2: Thiamine biomarkers in human milk and blood samples, by treatment group (in per-protocol population)[1]

		n	**Placebo (0 mg)**	***n***	**1.2 mg**	***n***	**2.4 mg**	***n***	**10 mg**
**Human milk total thiamine concentrations (µg/L)**[2]									
2 weeks postpartum		72	135.2 (72.4)	80	130.7 (73.0)	72	119.4 (62.1)	75	121.9 (74.6)
4 weeks postpartum		72	117.0 (55.9)	80	143.8 (55.5)	72	172.5 (77.3)	75	160.8 (72.2)
12 weeks postpartum		72	119.8 (62.0)	79	149.8 (59.8)	72	149.6 (54.5)	75	141.5 (57.7)
24 weeks postpartum		72	148.8 (73.3)	77	184.5 (78.1)	70	190.1 (86.2)	73	207.1 (77.8)
**Whole blood thiamine diphosphate (ThDP; nmol/L)**									
**Mother**									
2 weeks postpartum	Raw		72.9 (28.9)		69.0 (25.0)		68.6 (25.4)		71.2 (21.0)
	Corrected[3]	25	197.5 (63.5)	21	199.5 (58.5)	19	182.8 (78.3)	23	195.0 (50.6)
24 weeks postpartum	Raw		80.4 (21.8)		96.9 (22.9)		98.8 (21.7)		111.1 (25.2)
	Corrected[3]	71	217.2 (52.3)	78	261.1 (63.5)	70	260.2 (50.8)	74	297.2 (62.0)
**Infant**									
24 weeks postnatal	Raw		57.3 (14.1)		73.9 (17.2)		72.8 (19.5)		73.2 (17.4)
	Corrected[3]	66	178.5 (43.9)	73	232.3 (62.5)	66	220.7 (57.6)	72	224.0 (52.1)
**Erythrocyte transketolase activity coefficients (ETKac)**									
**Mother**									
2 weeks postpartum		71	1.14 (0.07)	80	1.16 (0.10)	72	1.17 (0.10)	75	1.17 (0.09)
24 weeks postpartum		71	1.21 (0.07)	77	1.15 (0.06)	67	1.14 (0.05)	74	1.12 (0.05)
**Infant**									
24 weeks postnatal		59	1.18 (0.07)	74	1.14 (0.05)	63	1.14 (0.05)	73	1.13 (0.06)

[1] Data are mean (SD) based on raw data.
[2] Human milk total thiamine concentrations are calculated as free thiamine + (thiamine monophosphate x 0.871) + (thiamine diphosphate x 0.707).
[3] Values corrected for hematocrit.

Online Supplementary Material:
Gallant et al., Low dose thiamine supplementation of lactating Cambodian mothers optimizes human milk thiamine concentrations and would be expected to prevent beriberi: a randomized controlled trial

Supplementary Table 3: Mean differences in human milk and blood thiamine biomarkers, between treatment groups (in per-protocol population)[1]

	**Unadjusted Mean difference (µg/L) (95% CI)**	**P[2]**	**Adjusted[3] Mean difference (µg/L) (95% CI)**	**P[2]**
**Human milk total thiamine concentrations (µg/L)[4]**				
1.2 mg – Placebo (0mg)	30.4 (15.0, 45.7)	<0.0001	31.1 (16.0, 46.2)	<0.0001
2.4 mg – Placebo (0mg)	41.0 (24.4, 57.7)	<0.0001	42.8 (26.5, 59.0)	<0.0001
2.4 mg – 1.2 mg	10.7 (-5.7, 27.0)	0.3	11.6 (-4.5, 27.7)	0.2
10 mg – Placebo (0mg)	38.4 (22.2, 54.7)	<0.0001	40.5 (24.6, 56.4)	<0.0001
10 mg – 1.2 mg	8.1 (-8.0, 24.1)	0.6	9.3 (-6.5, 25.2)	0.4
10 mg – 2.4 mg	-2.6 (-19.7, 14.5)	1.0	-2.3 (-18.9, 14.3)	1.0
Overall		<0.0001		<0.0001
**Whole blood thiamine diphosphate (ThDP; nmol/L)[5]**				
**Mother**				
1.2 mg – Placebo (0mg)	54.1 (10.9, 97.4)	0.008	53.2 (16.4, 90.0)	0.002
2.4 mg – Placebo (0mg)	45.4 (0.9, 89.9)	0.04	46.6 (9.1, 84.0)	0.009
2.4 mg – 1.2 mg	-8.7 (-55.0 37.5)	0.96	-6.6 (-45.3, 32.1)	0.97
10 mg – Placebo (0mg)	84.6 (42.4, 126.8)	<0.0001	80.8 (44.8, 116.8)	<0.0001
10 mg – 1.2 mg	30.5 (-13.6, 74.6)	0.28	27.6 (-9.5, 64.8)	0.21
10 mg – 2.4 mg	39.2 (-6.1, 84.5)	0.11	34.2 (-3.6, 72.1)	0.09
Overall		<0.0001		<0.0001
**Infant**				
1.2 mg – Placebo (0mg)	53.1 (29.2, 76.9)	<0.0001	54.5 (31.2, 77.9)	<0.0001
2.4 mg – Placebo (0mg)	42.1 (17.7, 66.5)	<0.0001	41.7 (17.8, 65.6)	<0.0001
2.4 mg – 1.2 mg	-10.9 (-34.8, 12.9)	0.6	-12.9 (-36.3, 10.6)	0.5
10 mg – Placebo (0mg)	44.7 (20.8, 68.7)	<0.0001	44.7 (21.2, 68.1)	<0.0001
10 mg – 1.2 mg	-8.3 (-31.8, 15.1)	0.8	-9.9 (-32.8, 13.1)	0.7
10 mg – 2.4 mg	2.6 (-21.3, 26.6)	1.0	3.0 (-20.5, 26.4)	1.0
Overall		<0.0001		<0.0001
**Erythrocyte transketolase activity coefficients (ETKac)[5]**				
**Mother**				
1.2 mg – Placebo (0mg)	-0.06 (-0.08, -0.03)	<0.0001	-0.06 (-0.08, -0.03)	<0.0001
2.4 mg – Placebo (0mg)	-0.07 (-0.09, -0.04)	<0.0001	-0.07 (-0.09, -0.04)	<0.0001
2.4 mg – 1.2 mg	-0.01 (-0.03, 0.02)	0.77	-0.01 (-0.03, 0.02)	0.75
10 mg – Placebo (0mg)	-0.08 (-0.11, -0.06)	<0.0001	-0.08 (-0.11, -0.06)	<0.0001
10 mg – 1.2 mg	-0.03 (-0.05, -0.00)	0.03	-0.03 (-0.05, -0.00)	0.02
10 mg – 2.4 mg	-0.02 (-0.04, 0.01)	0.33	-0.02 (-0.04, 0.01)	0.27
Overall		<0.0001		<0.0001
**Infant**				
1.2 mg – Placebo (0mg)	-0.04 (-0.07, -0.01)	0.0008	-0.04 (-0.07, -0.01)	0.0008
2.4 mg – Placebo (0mg)	-0.04 (-0.07, -0.02)	0.0004	-0.04 (-0.07, -0.02)	0.0004
2.4 mg – 1.2 mg	-0.00 (-0.03, 0.02)	1.0	-0.00 (-0.03, 0.02)	1.0
10 mg – Placebo (0mg)	-0.05 (-0.08, -0.03)	<0.0001	-0.05 (-0.08, -0.03)	<0.0001
10 mg – 1.2 mg	-0.01 (-0.04, 0.01)	0.5	-0.01 (-0.04, 0.01)	0.5
10 mg – 2.4 mg	-0.01 (-0.04, 0.02)	0.7	-0.01 (-0.04, 0.02)	0.7
Overall		<0.0001		<0.0001

[1] Data are mean differences (95% CI) based on analysis of raw data.
[2] Post-hoc (Tukey) adjusted p-values for multiple comparisons.
[3] Adjustments: Human milk concentrations: adjusted for human milk total thiamine at 2 weeks, and health center; ThDP: adjusted for health center (mother and infant) and maternal thiamine

diphosphate concentrations (corrected for hematocrit) at 2 weeks (mother only); ETKac: adjusted for health center (mother and infant) and erythrocyte transketolase activity coefficient at 2 weeks (mother only).

[4] Linear mixed effects model was used to analyze the data at 4, 12 and 24 weeks with a treatment by time interaction model. Mean differences between treatment groups collapsed across all time points (4, 12 and 24 weeks) are reported, as the treatment by time interaction p-value was >0.05 and hence was dropped from the analysis model.

[5] Linear regression was used to estimate the mean differences between treatment groups at 24 weeks.
